# Clinicians' perspectives of a care coordination model for children with medical complexity

**DOI:** 10.3389/fped.2025.1626439

**Published:** 2025-09-08

**Authors:** Emily E. Munn, Jake W. Griffin, Edmond Ramly, Mary R. Ciccarelli, Melissa M. Pangelinan

**Affiliations:** ^1^ School of Public Health-Bloomington, Department of Kinesiology, Indiana University, Bloomington, IN, United States; ^2^ School of Public Health-Bloomington, Department of Health and Wellness Design, Indiana University, Bloomington, IN, United States; ^3^ School of Public Health-Bloomington, Center for Health by Design, Bloomington, IN, United States; ^4^ Department of Pediatrics, Indiana University School of Medicine, Indianapolis, IN, United States; ^5^ Indiana School of Medicine, Stark Neuroscience Research Institute, Bloomington, IN, United States

**Keywords:** nurse navigator, healthcare efficiency, clinician burnout, healthcare quality, complex care

## Abstract

**Background:**

The Indiana Complex Care Coordination Collaborative (IC4) is a statewide initiative designed to enhance care for children with medical complexity (CMC) by embedding nurse care coordinators within clinical practices. This study explored clinicians' perspectives on how these coordinators influenced care delivery.

**Methods:**

Fourteen clinicians from six participating medical systems completed semi-structured interviews. Discussions focused on the impact of care coordinators on workflow, patient care, clinician workload, and the medical home experience. Transcripts were analyzed using an inductive approach to identify key themes and insights.

**Results:**

Clinicians consistently described care coordinators as central to improving communication and access for families, serving as a reliable point of contact, and facilitating smoother interactions with the healthcare system. They noted that care coordinators helped organize patient information, enabling focused and efficient clinical encounters. This support reduced administrative burden and allowed clinicians to prioritize patient needs more effectively. Additionally, care coordinators played a vital role in educating staff, advocating for families, and addressing both medical and non-medical concerns. While clinicians emphasized the value of care coordination, they also highlighted the need for clearer role definitions and adequate training to ensure coordinators are fully integrated and utilized appropriately.

**Conclusion:**

Clinicians reported that embedded care coordinators significantly enhanced the quality, efficiency, and responsiveness of care for CMC. Their perspectives underscore the importance of structured, well-supported care coordination to improve clinical practice and patient outcomes.

## Introduction

Children with medical complexity (CMC) are a group of pediatric patients who require substantial and consistent medical intervention (1, 2). Although CMCs comprise less than 5% of pediatric patients in the United States (US) (3, 4), they account for over a third of Medicaid spending (5). CMCs frequent the emergency room, have long inpatient admissions, and are commonly readmitted to hospitals, all of which increase their health care cost and negatively impact the child's and caregiver's quality of life (1). For this paper, CMC is defined as children with three organ systems involved, necessitating longitudinal care from at least three specialists (2).

For CMCs, the healthcare system is often fragmented and difficult to navigate due to numerous specialist visits and diverse needs (6). Caregivers of CMCs spend considerable time planning and coordinating healthcare for their CMCs (6, 7). Caregivers often report that the care workload fundamentally changes their identity (8), physical health (8), mental health (8–10), marriage/romantic relationship (8, 10), ability to care for other children (8, 10), employment (8, 10), financial stability (8, 10), time constraints (8), and social life (8). Healthcare providers have suggested that families often struggle to navigate the healthcare system independently (11).

Clinicians also report challenges. Primary care clinicians report a lack of skills and time to provide high-quality care for CMCs (11). Primary care clinicians have indicated that CMCs require more time, effective communication, and thorough planning to maintain high-quality care (12). With primary care clinicians and caregivers experiencing challenges, workload for coordinating specialty care and developing comprehensive, patient-centered care plans often falls through the cracks (11, 13). Clinicians and families can benefit from services focused on coordinating care for CMCs.

Nurses and social workers often serve as care coordinators to reduce the burden of care for clinicians and families by coordinating appointments, communicating with specialists, and collating resources from medical and non-medical providers (e.g., insurance companies, waiver case managers, community organizations, etc.). Our definition of care coordination includes the concept of “patient-centered care interventions”. As such, the patient and their family are included in shared decision-making and healthcare goal setting (14). Care coordinators collaborate with families and healthcare staff to schedule appointments, ensure adherence to care plans, facilitate access to medical supplies, provide support for medical and other services, and serve as liaisons between all parties (15). Families report that care coordination improves the quality of care (10, 15, 16). Moreover, care coordination also reduces costs to payers and out-of-pocket expenses incurred by families of CMCs (17–20).

Care coordination has been utilized outside the US to improve primary and specialized care through nurse-led programs, enabling greater access to clinical team members, comprehensive and patient-centered care planning, and appointment management (21, 22). While studies have shown the clear impact of care coordination on families and healthcare spending in the US and globally, less is known regarding the impact on primary and specialty care clinicians.

Previous research conducted to understand clinicians' perspectives has mainly focused on the sustainability of care coordination with a broad base of patients (23, 24). One study surveyed stakeholders, including clinicians, on their perspective of care coordination of hospitalized CMCs (25). This study indicated that parents, nurses, and clinicians highly value care coordination in this acute care setting; however, it did not include other settings.

Given the limited information on the impact of care coordination on clinicians working in primary and specialty practices, this study sheds new light on clinicians' experiences with care coordination for CMCs. This study examines a care coordination model developed and implemented by the Indiana Complex Care Coordination Collaborative (IC4).

## Methods

### Study design

We employed a qualitative descriptive study design to characterize care coordination from the clinicians' perspective (26, 27). Prior studies of clinicians' perspectives frequently use this qualitative design approach to describe how healthcare interventions function and provide insights into their implementation characteristics (e.g., acceptability, workflow, impact on the practice, etc (28–32). Qualitative descriptive studies close gaps in the understanding of a phenomenon by purposefully sampling key informants following naturalistic inquiry principles with “only a commitment to studying something in its natural state” to the extent possible, using data collection and analysis “techniques that allow the target phenomenon to present itself as it would if it were not under study (33, 34).” As such, the qualitative descriptive design starts with “no *a priori* commitment to any one theoretical view of a target phenomenon (35), while being open to using different theoretical views in the interpretation of data and findings. Qualitative study methods, data, and findings were reviewed by a qualitative research methodology expert with PhD training in human factors and post-doctoral training in implementation science (ER). The Indiana University Institutional Review Board reviewed and approved the study protocol.

### Setting

The Indiana Complex Care Coordination Collaborative (IC4) was launched as part of a Health Resources and Services Administration (HRSA) 10-state collaborative demonstration project. At the time of this study, the program was funded by a Home and Community-Based Services (HCBS) Stabilization Grant (CFDA # 93.778 Medical Assistance Program). Practices were invited to participate in the program between 2019 and 2024. Each health system hired a registered nurse whose salary was covered by the project grants. These nurses received six months of intensive training, including didactics, coaching, quality process reporting, and plan of care auditing (∼75 h of training), and subsequently joined a longitudinal community of practice for semi-monthly virtual meetings.

Each care coordinator enrolls 100 patients identified by each practice as eligible for the IC4 program. The care coordinator performs a medical record review and intake interview with the primary caregiver for each patient. Each practice includes a physician champion for the IC4 program. A shared plan of care is co-developed with the care coordinator and primary caregiver, which includes intake information and synthesizes a list of unmet needs and actions negotiated with each family to achieve the desired goals. The shared plan of care is vetted with the primary clinician for edits or suggestions and then uploaded into the medical record and distributed to specialists, the patient's payer organization, and other targeted recipients as the family desires. The care coordinator assists the family in task completion for goal achievement at a level of engagement that is determined by each family's need or desire for assistance. At office visits and at times when between-visit needs arise, the care coordinator updates the chart, verifies unmet needs with families, and sets new goals for the family in collaboration with the clinician. The care coordinator maintains contact with families at least quarterly, with full updates to the shared plan of care at least semi-annually. The care coordinator instructs families to use the usual office workflow processes for routine activities, such as scheduling a follow-up appointment or refilling a medication. While stepping in to facilitate unusual or complex needs, the care coordinator navigates prior authorizations for supplies, schedules multiple appointments on the same day, and more. They are also called upon to help share their knowledge in navigating systems of care with other clinical team members who provide services to other non-enrolled patients.

### Participants

Clinicians who participated in the IC4 program were recruited via email with an information letter that provided details about the study, protection of their identity, and confidentiality of their information. Ninety-four clinicians were invited from 14 practices across seven medical systems participating in the IC4 program. Clinicians from these practices had participated in the IC4 program for a minimum of 3 months (range 3–60 months). The clinicians from the medical system that most recently implemented IC4 (within 3 months) were not available to participate due to time constraints. Participants completed a demographic/information survey, which included questions such as the number of years in the medical field and the number of years working with a care coordinator.

### Data collection

We developed and pilot-tested a semi-structured interview guide, which is available as Supplementary Material. The interview guide was pilot-tested with three of the physician champions from the three clinics that have been part of the IC4 program since 2019. We specifically asked these physician champions if anything was missing from the interview guide or if they wanted to add any additional information about their experience with the IC4 care coordinators. The three physician champions stated that the interview guide was comprehensive. Therefore, no changes were made for the subsequent interviews, and at the end of each interview, the clinicians were asked if there was additional information they wanted to add about their experience with the IC4 care coordinators. The interview included 15 questions which examined five themes: the overall impact of care coordination, impact of care coordinator on patient care and needs, impact of care coordinator on the clinician, impact of the care coordinator on the practice, and impact of care coordinator on patient advocacy and education. Questions were followed by clarifying questions based on participant responses.

Individual semi-structured interviews were conducted by two PhD-trained researchers with graduate coursework in qualitative methods (EM & MP). The interviews were conducted remotely via video conference (Zoom™) to increase the transferability of findings by reducing geographic barriers and including a range of perspectives, as clinicians were dispersed across clinics within a 200-mile radius. The researchers referred to each participant by their pseudonym during the interview to ensure confidentiality. The interviews were recorded with the participants' consent. The interviews were recorded and transcribed through Zoom's built-in recording and automatic transcription software features. A research assistant reviewed the transcripts for errors and made necessary edits. All data was collected within 2 months of the initial invitation.

### Data analysis

We conducted a thematic analysis following six steps (36), applying an inductive approach for the coding step. Specifically, we used the open-coded and respondent/data-based meanings to guide the codes and extracted themes. Using Braun and Clarke (36) method for conducting thematic analysis, six phases of thematic analysis were implemented: (1) familiarizing yourself with your data (EM & JG), (2) generating initial codes (JG), (3) searching for themes (EM & JG), (4) reviewing themes (EM, ER, JG & MP), (5) defining and naming themes (EM, ER, JG & MP), and (6) producing the report (EM, ER, JG & MP). NVivo 14 (Version 14.24.2) software was used to facilitate the creation of the codebook, manage the initial coding, conduct inductive thematic analysis, and identify quotes. For each question, the analytic process was: (1) read the transcript, (2) chunk the response into smaller sections (performed by NVivo), (3) code each of the smaller sections, (4) identified board themes, (5) consolidate (NVivo) and define the themes. We planned to solicit more responses had we not arrived at data saturation, defined as the point at which no new themes emerged with the addition of data from more subjects. We employed established strategies to address rigor and trustworthiness in qualitative health services research, in general (37), and thematic analysis, specifically (38). We employed iterative consensus-building discussions in the code and theme phases, utilizing triangulation among researchers and seeking disconfirming evidence to enhance credibility and confirmability. We used data archiving, creation of an audit trail, and skeptical peer review across all phases to increase dependability and confirmability. To increase transferability, we complemented our sampling and data collection strategies with thick descriptions of context and findings. For each section of the results, the themes are reported in order from the theme with the most participants to endorse to the theme with the fewest participants.

## Results

Sixteen clinicians (13 in primary care, 3 in specialty practices) from six of the seven medical systems contacted the researchers and completed the demographic/information survey. Fourteen completed the 1-hour semi-structured interview and were included in the study. Two participants did not complete an interview due to schedule constraints or a lack of response after the initial email exchange. No new themes emerged from the interviews after 12 clinicians were interviewed. The demographics and basic information for the study participants who completed the study are listed below in Table 1.

**Table 1:** Clinician characteristics—sorted by Age and Sex.

Pseudonym	Age	Years as Clinician	Years at Current Practice	Years with IC4 CC	Previous care coordination program before IC4?	Race	Ethnicity	Sex
Kathy	49	18	2	<1	Yes	White	Not Hispanic or Latino	Female
Alex	35	6	2	<1	No	White	Not Hispanic or Latino	Female
Grace	71	39	36	5	No	White	Not Hispanic or Latino	Female
Sandra	36	5	5	<1	No	Multiple	Not Hispanic or Latino	Female
Jacobs	33	3	3	5	Unknown	White	Not Hispanic or Latino	Female
Charlie	44	7	7	3	Yes	White	Not Hispanic or Latino	Female
Annie	43	12	4	2	Unknown	White	Not Hispanic or Latino	Female
Jessica	42	8	8	2	No	White	Not Hispanic or Latino	Female
Andrew	36	3	2	1	No	White	Not Hispanic or Latino	Male
Harry	53	30	4	4	Yes	Asian	Not Hispanic or Latino	Male
Jennifer	49	13	13	1	Yes	White	Not Hispanic or Latino	Female
Sydney	58	30	26	<1	No	White	Unknown/Not Reported	Female
Cameran	65	40	30	3	No	White	Not Hispanic or Latino	Female
Laura	40	14	11	5	No	White	Not Hispanic or Latino	Female

The sixteen clinicians ranged in age from 33 to 71 years (*M* = 26.7). Their years of experience as clinicians ranged from 3 to 40 years (*M* = 16.3). Of the sixteen, four had previous experience working with a care coordinator before joining the IC4 program. Their time in the IC4 program ranged from about 6 months to 5 years.

**Table 2:** Major themes and subthemes.

Theme	Description	Clinicians Reporting
Major Theme 1		
*Sub Theme 1.1*	*Care coordinators open communication channels with care coordinators guided patients/families.*	14
*Sub Theme 1.2*	*Care coordinators bridged relationships with clinic staff and patient families*	14
*Sub Theme 1.3*	*Care coordinators improved access to specialists and care between visits.*	12
*Sub Theme 1.4*	*Care coordinators organized and prioritized care based on patient needs.*	12
*Sub Theme 1.5*	*Care coordinators enhanced patient accountability and care transitions.*	10
*Sub Theme 1.6*	*Care coordination services reduced their patient and caregiver stress.*	8
Major Theme 2		
*Sub Theme 2.1*	*Care coordinators streamlined access to information and care processes.*	14
*Sub Theme 2.2*	*The co-created (Family and care coordinator) Shared plans improved access to relevant medical data.*	7
*Sub Theme 2.3*	*Care coordination reduced clinician workload and brought team relief.*	7
*Sub Theme 2.4*	*Care coordinators helped with appointment scheduling and re-scheduling appointment*	6
*Sub Theme 2.5*	*Care coordinators increased efficiency, improve time constraints and mitigated burnout.*	5
Major Theme 3* *		
*Sub Theme 3.1*	*Educating clinicians and other staff*	11
*Sub Theme 3.2*	*Care coordinators directly support the other staff members within the practice*	11
*Sub Theme 3.3*	*Care coordinators’ specialized knowledge and advanced nursing skills were vital to the practice’s success.*	9
*Sub Theme 3.4*	*Care coordinators’ advanced training allows them to help educate clinicians and other staff at the clinic about complex medical conditions and available resources*	7
*Sub Theme 3.5*	*Care coordinators’ specialized knowledge of Medicaid and insurance waivers is valuable for patients' families and the clinic’s staff. *	7
Major Theme 4		
*Sub Theme 4.1*	*Addressing patient needs*	12
*Sub Theme 4.2*	Care coordinators act as advocate for patients and families, providing the clinicians with insights on the patient’s needs	9
*Sub Theme 4.3*	Due to care coordination, patients’ needs were more visible	8
*Sub Theme 4.4*	Patients were more comfortable with CC than clinicians and gave them more information, allowing them to be more comfortable sharing their needs.	3
*Sub Theme 4.5*	Care coordinators help provide holistic care by helping patients and families address medical and non-medical needs	12
Major Theme 5		
*Sub Theme 5.1*	*Improvements*	10
*Sub Theme 5.2*	*Care coordinators need a clearer role definition and onboarding needed based in each clinic.*	10
*Sub Theme 5.3*	*Care coordinators need more training on advocacy and communication.*	10
*Sub Theme 5.4*	*Concerns about the potential for blurred boundaries between patients and clinics and between clinicians and care coordinators due to the care coordinator's communication style and the role of social support*	5
*Sub Theme 5.5*	*“Head Nurse” style role in care coordination that they could ask questions and have on-going assistance*	2
Major Theme 6		
*Sub Theme 6.1*	*Care Coordinator Qualifications*	7
*Sub Theme 6.2*	*Adaptable to clinic needs.*	7
*Sub Theme 6.3*	*Excellent communication*	6
*Sub Theme 6.4*	*Experienced*	5
*Sub Theme 6.5*	*Caring and friendly*	4
*Sub Theme 6.6*	*Trustworthy and reliability*	2
Major Theme 7		
*Sub Theme 7.1*	*Shared Plan Workflow*	7
*Sub Theme 7.2*	*Clinicians reported that the shared plan of care was more documentation but very descriptive*	7
*Sub Theme 7.3*	*Requested that the shared plan of care be updated so that it populated with other medical records.*	4

### Theme 1: care coordinator role as a single point of contact

Clinicians reported that care coordinators provided their patients and caregivers with open lines of communication to answer time-sensitive questions or problems quickly, thereby reducing caregiver stress and unnecessary emergency department utilization. The care coordinators provided guidance for health and wellness, and the follow-up needed to achieve the patient's health goals. The care coordinators helped the patient and caregiver become more accountable and proactive in their health care planning, including engaging in transition planning early.

“I think it [care coordination] is invaluable for these families. Just that easy connection to have their questions answered, rather than going to the ER or letting something go or trying to figure out who to access at [the hospital] … it saves them time and stress. It saves their jobs. I feel like you know, and it helps them learn, too… keeping them out of the ER.” - Karen

“So I have a 12 or 13 year old (patient). We're talking about transition. We're talking about adult care… I think it's (care coordination) been helpful, in talking about some of those proactive things really early.” - Charlie

Reciprocally, care coordinators increased clinicians' ease and access to patients. The care coordinators helped clinicians organize and prioritize medical plans to best meet the patient's needs. They also served as a bridge to improve patient and clinic staff relationships and scheduling.

“The biggest improvement or game changer I've seen so far is actually access to me. My complicated patients being able to send a direct message to the care coordinator. I'm able then to bypass the 15 other people that are supposed to be gatekeeping me and say, yes, I do want to triple book myself to see this patient, because that's what they need” -Alyssa

Concerning the care coordinator's role managing care across practices (e.g., primary and specialty care), clinicians reported they believed it was easier for the families to access medical care, specialists, and primary care facilities. This access reduced caregiver workload and enabled coordinated communication among medical teams.

### Theme 2: care coordinator's effect on efficiency and workload

The care coordinators co-created the shared plan of care with the patients and caregivers, which focused on the most relevant medical information and provided up-to-date notes to streamline visits. By reducing the time spent reviewing the full medical record, clinicians could focus their limited time on high-priority needs and providing holistic care. The care coordinator's role in documentation, follow-up, and scheduling provided a sense of relief for clinicians and staff.

“The information is handed to me on a silver platter by the care coordinator. So I can spend more of my time providing higher level care for the patient, instead of being a secretary” - Alyssa

“I think that it's just extremely difficult to really maintain timing for patient and understand, you know how much time we have with them. I have 20 min to have with you. And so, I have to get through this, this, this and this, it's nice to know that somebody's gonna step in and be able to fill in some of those blanks.” – Charlie

“I think it's brought a sense of a little bit of relief to our team.” – Karen

### Theme 3: care coordinator role in educating clinicians and other staff

Clinicians reported that the care coordinator's specialized knowledge and advanced nursing skills were vital in supporting clinicians and other staff. They educate clinicians and other staff about complex medical conditions and collate available medical, community, and insurance resources (e.g., Medicaid home and community waivers).

“They had never applied for that Medicaid waiver… that came up in the middle of our was able to take the time to fill out all that paperwork.” - Annie

### Theme 4: care coordinator's role in addressing patient needs

Clinicians reported feeling more comfortable with the care coordinator, compared to clinicians and other staff, which enabled greater information sharing. Care coordinators enabled patients and caregivers to prioritize medical and non-medical needs, including transportation, diapers, community resources, connecting with other families, and assistance with medical devices. The care coordinators advocate for patients and families, providing clinicians with insights and prioritizing the patients' unique needs and priorities. The clinicians reported that they were better able to provide holistic care for the patients and caregivers.

“I think families are now a little more informed and understand that they can request different things. You know. They can feel comfortable doing that, and then they can get their needs met. Whereas before, you know, maybe they just kind of kept it to themselves.” - Charles

“The questions that she's asking.. They show they are people and not just patients.” - Kat

### Theme 5: care coordinator's scope of work

To improve the integration and onboarding of care coordinators into the medical home, clinicians reported that training should help define the care coordinator's role and scope of work for each practice. By including clinicians and clinic staff in training, the entire medical home will know how to leverage the unique knowledge and skills the care coordinators provide and improve operational efficiency. Moreover, a distinct title (e.g., “Head Nurse”) would enable others in the practice to feel comfortable asking questions and learning from the care coordinator.

“I don't exactly know where my role really should end, and .. a care coordinator's role should really take over.” - Jacobs

Clinicians raised concerns about the responsivity of care coordinators to patients' questions and needs via the direct messaging portal, which may blur boundaries (answering questions anytime) and responsibilities (requesting support outside the care coordinators' purview). Clinicians expressed concern about the care coordinators being overloaded with their large caseload (100 patients with complex needs per care coordinator). The clinicians also commented about expanding patient inclusion for the IC4 program (e.g., at least three subspecialists involved in longitudinal care) and starting at birth for some conditions, which would improve outcomes for more patients.

“I think the hard pieces is specifically at my clinic; many families do not have typical insurance or Medicaid. And so they're not qualified… they would also really benefit from the program” - Sandra

### Theme 6: care coordinator qualifications

Clinicians report that care coordinators were highly skilled nurses who were trustworthy, experienced, personable, adaptable, reliable, had excellent communication skills, and truly cared about their patients and families.

“Well, she's really good about communicating like between visits. If things come up she'll just shoot me an email for me to respond to and we do that shared plan of care. So, I'm always reviewing those. She's forwarding those to me on a regular basis.” - Charles

“I think that she really cares about the families, and that comes through. And that's really really great.” - Annie

### Theme 7: shared plan of care workflow

Clinicians reported that although the shared plan of care was descriptive and helpful, they found the current format and data entry cumbersome. They recommended that some information in the shared plan of care could be automatically populated and updated from the medical records, reducing the need to update two databases.

“The SPOC does not populate in the EMR. So it's a separate document that we're looking at in addition to our EMR chart for them. So that does make that a little cumbersome.” - Charlie

**Figure 1:**
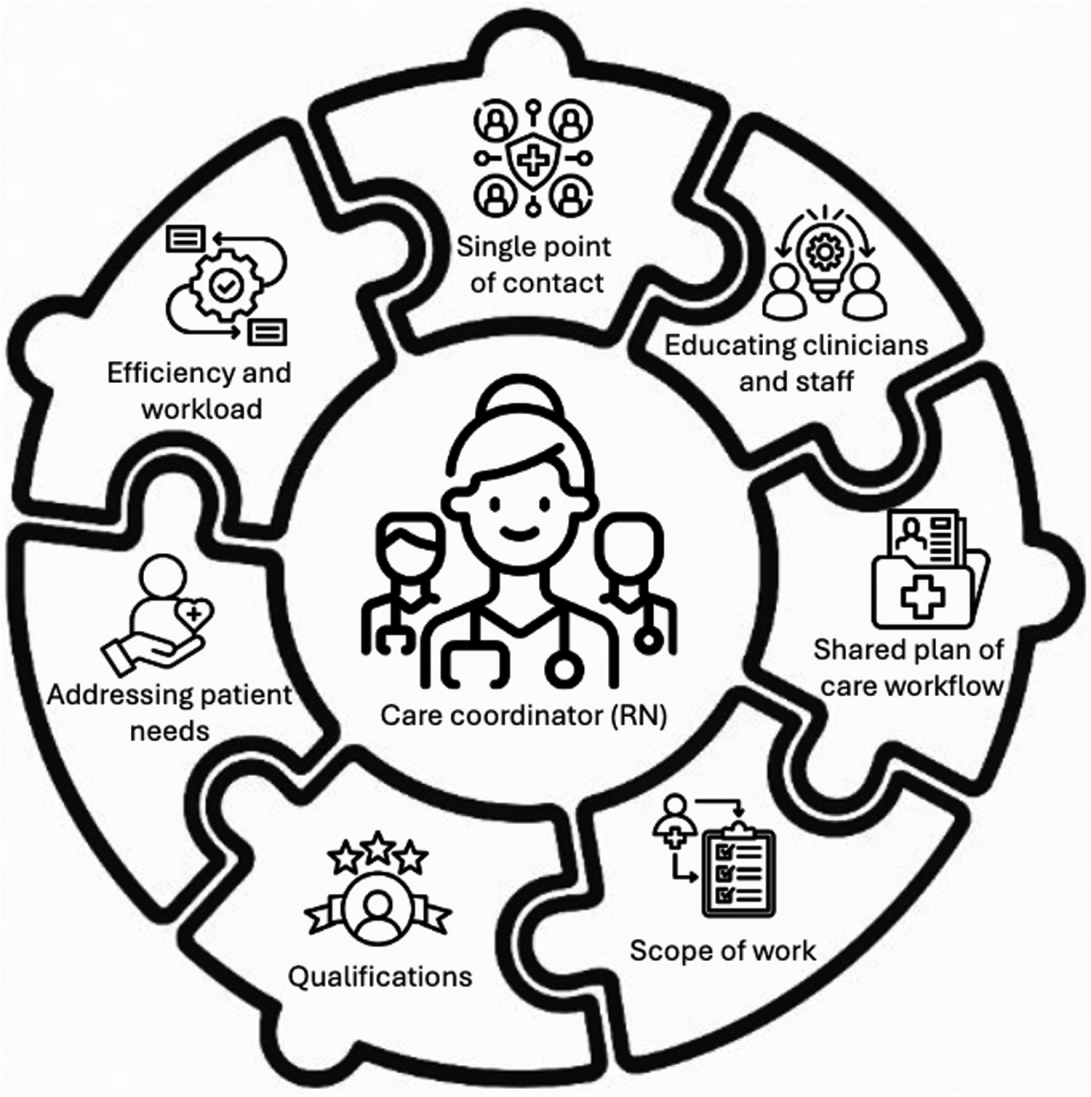
Infographic of key themes.

Figure 1 displays a summary of the major themes.

## Discussion

This study contributes to the growing body of literature on care coordination for CMCs by offering a nuanced understanding of clinicians' perspectives in both primary and specialty care settings. In contrast, previous research has emphasized caregiver experiences (10, 39–41), financial implications (17, 42), and hospital-based clinician viewpoints (25).

This model of care coordination embeds a registered nurse (RN) care coordinator within the primary or specialty care team, providing care for a targeted registry of complex patients. This approach not only meets the multifaceted needs of families but also alleviates clinician burden; a finding consistent with Foster et al. (12), who noted the disproportionate time demands of CMCs on providers. Clinicians reported that this model improved access to and quality of care, echoing caregiver reports in prior studies (39, 40). With two of the major stakeholders of care coordination reporting its significant benefit, care coordination for CMCs represent a worthwhile investment for insurance and other stakeholders.

### Core functions of care coordination

Care coordinators helped families navigate the healthcare system, reducing stress and improving their overall experience. Caregivers of CMC are tired, overwhelmed, and out of time (8, 39). Clinicians have previously reported deep concern for the ability of families of CMC to navigate the health systems (11).

Indeed, clinicians in this study report a significant benefit of care coordination in reducing caregivers' overall stress and medical care workload. CMCs need a primary care group that they are comfortable with that can communicate with specialists (13). Care coordinators can help to bridge the gap between primary care providers and patients. For example, care coordinators streamlined communication between clinicians and families (25) and served as a single point of contact to facilitate timely access to services across primary and specialty care. These serve to reduce stress previously reported by both caregivers (8, 9) and clinicians (11). Clinicians reported that caregivers felt more comfortable and supported, resulting in improved adherence to care plans.

Access to insurance waivers, transportation, appointment schedules, reminders, and follow-ups provided by the care coordinator enabled thorough wrap-around care. Again, addressing these unmet needs reduced caregivers' feelings of being overwhelmed and stressed and provided peace of mind for the clinicians (Munn et al., Under Review). In particular, the co-developed shared care plan emerged as a critical tool, offering clinicians a holistic view of the child and family enabling person- and family-centered care (43, 44). Indeed, clinicians reported the ability to shift their clinical lens from a purely medical focus to a broader understanding of patient and family social and emotional needs. Clinicians reported that this approach enhanced their ability to deliver comprehensive care and strengthened relationships with families.

Clinicians report a lack of training in caring for CMCs (13). Care coordinators were crucial in educating clinicians and other staff about complex medical conditions and available resources within this project. All IC4 care coordinators were registered nurses (RN) and received additional specialized training for working with CMC. In comparison, most nursing staff in participating practices are medical assistants (MAs) and licensed practical nurses (LPNs). The value of another RN on staff, in addition to their care coordination training, provided advanced knowledge and nursing diagnostic skills to care for the layered complexities of CMC is clear. Clinicians reported that other members of staff (MAs, etc.) would go to the care coordinators to ask questions about various processes and services. Their specialized skills spread across the practice to other children not qualifying for care coordination. Care coordinators are valuable, highly trained nurses who are compensated at a level that helps to retain them in their workplace. With a shortage of healthcare workers, maintaining the highest-quality workers is critical.

### Care coordination challenges and future program directions

Clinicians indicated several challenges to the overall success of care coordination, including the need to define the role of care coordinators to others on the team and the care coordinator's role regarding others' roles in the practice. A separate technical assistance center provides care coordination training for RNs to become care coordinators. This approach creates a level of separation between the training and the work environment. Despite existing structured activities to orient the care coordinator and the practice team to the program, further modifications and/or updates may be warranted to better integrate and embed the care coordinators within each practice. However, concerns regarding integration were nominal compared to clinicians' satisfaction with the program. They wanted to expand these services to more patients, ideally starting as soon as complex needs are identified. This program was funded over six years as a Medicaid demonstration project, and both sustainability and expansion depend on the implementation of payer models for value-based care. More work is needed to demonstrate the correct workload per nurse and to provide proof of cost-effectiveness to instigate payer action.

### Study limitations and future research directions

While this study focused on a self-selected group of primary and specialty care physicians in the Midwest US, all of whom participated in the same care coordination program, this sampling approach was a deliberate methodological decision aligned with the study's qualitative aims. The participant pool, primarily female and white, reflects the demographics of the program and region. While this may limit generalizability, it provides valuable insight into the lived experiences of clinicians within this specific context.

The absence of quantitative data is not a limitation, but rather a consequence of the study's qualitative design, which sought to explore nuanced perspectives and experiences that are not easily captured through numerical measures. Clinicians noted the need for more tailored training for care coordinators, suggesting that future studies should examine implementation strategies and training protocols across diverse clinical settings. Additionally, while this study identified perceived impacts on clinician stress, longitudinal research is needed to assess the long-term effects of care coordination on clinician burnout and patient outcomes.

These findings suggest that embedding RN care coordinators within outpatient teams is a feasible and effective model that other healthcare settings can adopt to improve care for CMCs.

Future research could benefit from mixed methods approaches to align qualitative findings with existing quantitative data and to explore quantifiable variables such as time saved through care coordination.

## Data Availability

The raw data supporting the conclusions of this article will be made available upon request to the corresponding author (emmunn@iu.edu).
